# Critical Points of Risk in Registered Nurses' Use of a National Early Warning Score—Perceptions and Challenges

**DOI:** 10.1111/jan.16656

**Published:** 2024-12-06

**Authors:** Claire Nadaf, Suzanne Bench, Yvonne Halpin, Louise Terry

**Affiliations:** ^1^ Health Sciences University Bournemouth UK; ^2^ London South Bank University London UK

**Keywords:** clinical judgement, deteriorating patient, early warning systems, hermeneutic, NEWS, patient safety, phenomenology

## Abstract

**Aim:**

To explore Registered Nurses' experiences and perceptions of using the National Early Warning Score in the U.K. as part of the recognition and management of acute adult patient deterioration.

**Design:**

Hermeneutic Phenomenological design.

**Methods:**

Sixteen Registered Nurses from a U.K. NHS hospital were interviewed using an interpretative phenomenological approach (2019–2020).

**Results:**

Registered Nurses' use of NEWS highlighted 3 risk areas: delegation of vital sign monitoring to unregistered staff leading to uncertainty and delayed escalation, junior nurses' over‐reliance on NEWS and deference to expertise, and senior nurses' self‐management of deteriorating patients. The workplace culture revealed frequent compromises and limited learning opportunities.

**Conclusion:**

When using NEWS, failure to recognise associated risks threatens patient safety. Wrong decisions at the three ‘pinch points’ may lead to missed chances in preventing deterioration. Incorrect judgements may lead to unrecognised patient deterioration or inappropriate management leading to preventable adverse events.

**Implications for the Profession and Patient Care:**

The way in which NEWS is used by healthcare professionals brings inherent patient safety risks. Addressing education gaps and fostering a supportive culture in nursing, valuing and enhancing nurses' clinical judgement, is crucial for mitigating these risks and ensuring patient safety.

**Impact:**

The study deepens understanding of nurses' use of NEWS and identifies components affecting the recognition of patient deterioration.

**Reporting Method:**

Adherence to the EQUATOR guidelines SRQR confirmed.

**Patient or Public Contribution:**

Service user involvement was included within the design of the study and ethical approval.

**Summary**

- What does this paper contribute to the wider global clinical community?
  ○ Greater understanding of the use of a National Early Warning Score by nurses which potentially threaten patient safety.
  ○ Evidence that use of Early Warning Scores may limit the development of clinical judgement skills in junior nurses.

## 1 Introduction

Undeniably, recognising deterioration remains one of the predominant areas of patient safety concern globally. Acute deterioration is a time‐critical situation as failure to detect deterioration or delay in acting can lead to adverse events such as unplanned admission to a critical care unit or death (Smith et al. 2013; Chua et al. 2017). A deteriorating patient is defined as “A patient that moves from one clinical state to a worse clinical state which increases their individual risk of morbidity, including organ‐dysfunction, protracted hospital stay, disability or death” (Jones et al. 2013, 1031). The deteriorating patient phenomenon, also frequently referred to in the literature as ‘Failure to rescue’ (FTR) first emerged in the United States of America (U.S.) and has been subject to international debate since the 1990s, when it was recognised as posing a significant patient safety risk. In the U.S., FTR rates are a patient safety indicator and metric of hospital quality and institutional competence (Mant 2001). Root cause analysis has identified three critical stages of FTR including failure to recognise complications, failure to relay information, and failure to react (Burke, Downey, and Almoudaris 2020).

Internationally, patient safety has increasingly been in the public eye, with growing public awareness of adverse events through the media and campaigns for improvement, such as the international Surviving Sepsis Campaign (Evans et al. 2021). The focus on patient safety in healthcare dates back to the 1990s, attributed to the work of Reason (1990), followed by studies on clinical error and quality improvement initiatives (Donaldson, Appleby, and Boyce 2000). Prior to this, there was a perceived acceptance of avoidable harm caused by health professionals as a consequence of healthcare delivery (Vincent 2010).

A prominent 2012 study (Hogan et al. 2012), that subsequently formed the basis of several improvement initiatives, reported that 5.2% of hospital deaths had at least a 50% chance of being preventable in the United Kingdom (U.K.). This represents 11,859 adult preventable deaths in the National Health Service (NHS) each year. Of those preventable deaths, 31% were attributed to poor monitoring. Subsequent reports have continued to identify problems in recognising and responding to deterioration producing sub‐optimal care (Healthcare Safety Investigation Branch 2019); however, recent national data reporting deaths attributed to poor monitoring is not available.

Numerous global improvement initiatives have attempted to combat this phenomenon to decrease adverse events and improve patient outcomes (Burke, Downey, and Almoudaris 2020). One such initiative is Early Warning Systems (EWS), also referred to as track and trigger systems (TTS), introduced in late 1990s, to reduce unnecessary deaths by predicting patient deterioration through physiological signs triggering warnings. EWS are powerful tools based on clinical prediction models, implemented globally with multiple variations in place (Gerry et al. 2020) such as Modified Early Warning Scores (MEWS), Vitalpac Early Warning Score (VIEWS), Individual Early Warning Score (I‐EWS), and National Early Warning Score (NEWS). The lack of underpinning evidence for the efficacy of these TTS has been highlighted alongside concerns around their variable sensitivity (Gerry et al. 2020). Whilst a large amount of existing research supports the ability of a range of EWS to predict both patient mortality and admission to a critical care unit (Smith et al. 2013) empirical data suggest that their use in clinical practice has not greatly improved early detection and recognition of patient deterioration (NHS England 2018), with limited evidence of their impact on patient outcomes.

Vital signs measurement is central to the use of EWS. Across the world, nurses play a central role in this important aspect of healthcare practice, undertaking patient monitoring. Research into nurses' use of various EWS has expanded with more recent evidence considering reliance on tools (Massey et al. 2024) and the impact on critical thinking, clinical reasoning and judgement (Le Lagadec et al. 2024). This study aimed to add to the body of existing evidence by exploring nurses' experiences and perceptions of using NEWS.

## 2 Background

Preventable deterioration falls within the realm of patient safety initiatives which have emerged from a wide set of influences including aviation which is widely recognised as a safety critical industry. Systems approaches to patient safety are frequently proposed in healthcare, predicated on the premise that well‐designed systems prompt individuals towards desirable behaviour and restrain them from undesirable ones (Clarkson et al. 2018). However, systems approaches need to be underpinned by an understanding of the vital link between humans and healthcare systems in helping to reduce medical errors and improve patient safety (Leape 2004).

One such system is the Rapid Response System (RRS), developed to proactively identify patients at risk of clinical deterioration, placing patient safety at the heart of the system. The concept of the RRS was first introduced in Australia and the U.S. in the mid 1990s with other countries implementing RRS following recommendations of the Institute for Healthcare Improvement in their Five Million Lives Campaign (2006). The RRS includes an afferent and an efferent arm. The afferent arm is the detection arm in which the process of recognition of deterioration takes place. The efferent arm refers to the management of the patient by the response team. Sitting within the afferent arm of an RRS are EWS. EWS have been implemented globally with multiple systems in place (Royal College of Physicians 2017).

EWS consist of two steps. Step one involves monitoring vital signs, plotted on an observation chart, assigning scores to parameters like respiration rate, oxygen saturations, and blood pressure. Higher scores signify greater deviation from normal. Step two includes a clinical response protocol based on trigger thresholds, guiding users on recommended actions based on overall scores. In an aggregated system, scores from each parameter are combined to give an overall score. Nurses, who are responsible for vital sign monitoring, are central to the effective use of EWS.

Studies across a range of EWS report efficacy in identifying deterioration and predicting patient outcome such as unplanned intensive care admission (Chua et al. 2017). Systematic reviews have highlighted the impact of cultural, organisational, and educational factors on identification of deterioration (Ferguson, Baldwin, and Henderson 2024; Chua et al. 2017). More recently there has been an increase in studies focused on nurses as the main users of EWS reporting that despite EWS being considered a helpful risk assessment tool (Langkjaer et al. 2023; Le Lagadec et al. 2024) which offers a shared language between healthcare professionals (Langkjaer et al. 2021). Concerns exist around poor compliance (Massey et al. 2024); heavy reliance on EWS and the implications on critical thinking (Le Lagadec et al. 2024). De‐personalisation of care (Ferguson, Baldwin, and Henderson 2024) has also been associated with the inflexibility of the NEWS tool.

Concern over the use of multiple EWS led the U.K. to develop a standardised EWS, the National Early Warning Score (NEWS) in 2012 with a subsequent update in 2017 to NEWS2 (Royal College of Physicians (RCP), 2017). NEWS is an aggregated system based upon a logistic regression model, developed by consensus from a working party (Gerry et al. 2020). Need for further evaluation of the use of NEWS was highlighted in 2019 by the HealthCare Safety Investigation Branch who cited a reliance on NEWS which may offer false reassurance to healthcare staff. Existing studies show that the implementation of NEWS has not eradicated missed patient deterioration (Healthcare Safety Investigation Branch 2019), and while several recent studies have discussed the use of EWS, few have focused on NEWS and its use by nurses.

## 3 The Study

The aim of the study was to explore Registered Nurses' (RN) experiences and perceptions of using NEWS in the U.K. as part of the recognition and management of acute adult patient deterioration.

Objectives were:

- To explore nurses' experiences and perceptions of using NEWS in the recognition and management of acute deterioration in adult patients.
- To identify elements that influence nurses' use of NEWS in the clinical area.
- To develop a deeper understanding of the interaction between NEWS and nurses' clinical judgement and decision‐making.

## 4 Methods/Methodology

### 4.1 Design

This qualitative study utilised a hermeneutic phenomenological approach, underpinned by Gadamerian philosophy, exploring RNs experiences and perceptions of using NEWS through in‐depth interviews. Phenomenology is considered to be one of the foremost philosophies that guide knowledge generation in nursing (Moi and Gjengedal 2008) with researchers focused upon gaining insight and developing understanding into lived experience. Hermeneutics is the science of interpretation, the study of understanding to decipher meaning, with hermeneutic understanding requiring an interpretative or translational stance (Dibley et al. 2020). Researchers applying hermeneutic phenomenology are not attempting to seek a final or absolute truth (Gadamer 2004) but to present the experiences of individuals interpreting the meanings, provoking thinking, awareness and understanding.

Central to the methodology is the concept of a fusion of horizons, an understanding that mediates between people and experiences (Gadamer 2004). This is a point where personal perspectives are acknowledged and may be altered leading to a changing of perspectives as a critical part of the research process. The fusion of horizons occurs when the interpreter's horizon intersects with the horizon of another (such as the interviewee) or the context of the text (whether verbal or written) being examined, expanding both horizons. To enable this, the researcher undertakes an examination of propositions as part of the process of understanding the lived experience of each participant, constantly considering their own thoughts in relation to the dialogue with participants and impact of this on the findings.

Integral to the hermeneutic phenomenological approach is the Hermeneutic circle or spiral, a continuous action where the researcher goes backwards and forwards or in a circular motion to gain deeper understanding of the phenomena and interpretation of the lived experience. The circle is represented by the notion that understanding does not have an end, there is no final or absolute truth (Gadamer 2004).

### 4.2 Theoretical Framework

Tanner's (2006) model of clinical judgement provided a theoretical underpinning for this study. Tanner (2006) proposed four stages in the process of clinical judgement: noticing, interpreting, responding, and reflecting. Each stage requires understanding and knowledge to appreciate the characteristics of the clinical situation and an appropriate response within a given timeframe, recognising the impact of experience, context, and relationships on the process, reflecting underlying theories around clinical decision‐making. Tanner (2006) understood that clinical judgement is influenced by many variables including how familiar the nurse is with the patient and their pattern of response to nursing interventions. This resonates with Benner's (2001) work recognising the impact of experiential learning on judgement. Table 1 provides an overview of the stages of clinical judgement in application to the perceived action, knowledge, and skills in relation to the care of a deteriorating patient.

**TABLE 1:** Application of the four stages of clinical judgement (Tanner 2006).

Stage of process	Action	Skill/Knowledge required
Noticing	Vital Sign monitoring + NEWS Observation of soft signs Patient report of changes	Clinical skills/non‐clinical skills Background knowledge of scenario Contextual knowledge Knowing the patient
Interpreting	Analysis of findings Reasoning processes Intuitive processes Pattern Recognition	Applied anatomy and physiology. Knowledge of causes of deterioration
Responding	Further Assessment beyond NEWS such as ABCDE Initial nurse‐led intervention that is, medication administration, oxygen administration Escalation to senior/expert	Clinical skills for systematic assessment Applied anatomy and Physiology. Clinical Guidance Communication
Reflecting	Reflecting in action Reflection on action	Ability to read the patient and response to the intervention. Skills of reflection to identify clinical learning. Recognising outcomes

### 4.3 Study Setting

The study setting was a single site acute NHS hospital in London, England, serving a population of over 300,000 people, with more than 500 beds and directly employing over 2500 staff. The hospital had implemented the NEWS2 tool following its release in 2017. A Critical Care Outreach Team (CCOT) was available 24‐h a day, 7 days a week.

### 4.4 Participants

A purposive sampling approach identified RNs who used NEWS, working in permanent roles in acute adult in‐patient wards within the hospital. Non‐permanent RNs or those working in speciality areas such as Critical care were excluded from the study. This approach to sampling involved the identification and selection of individuals with knowledge or experience of the phenomenon of interest (Table 2). Data collection continued until a wide range of experiences had been collected to answer the research question and therefore attaining data saturation.

**TABLE 2:** Sample demographics.

Participant ID	Total Years as an RN	Year since registration with the U.K. NMC^a^	Year since registration Overseas (if applicable)	AFC Band^b^	Clinical area
N1	12	9 months	12 years	5	Medical
N2	10	18 months	10 years	5	Medical
N3	5	1 year	5 years	5	Medical
N4	41	41 years	Not applicable	6	Mixed medical/surgery
N5	14	14 years	Not applicable	7	Respiratory
N6	25	25 years	Not applicable	8a	Resus Lead
N7	25	25 years	Not applicable	8a	Acute admissions
N8	12	5 years	12 years	7	Stroke
N9	5	5 years	Not applicable	5	Acute admissions
N10	12	12 years	Not applicable	5	Stroke
N11	8	3 months	8 years	5	Stroke
N12	5	4 years	5 years	7	Orthopaedic
N13	10	3 years	10 years	6	Surgical
N14	< 1 year	1 year	Not applicable	5	Respiratory
N15	< 1 year	1 year	Not applicable	5	Acute admissions
N16	8	4 years	8 years	6	Surgical

^a^ NMC –The Nursing and Midwifery Council is the regulator for nursing and midwifery professions in the U.K.

^b^ AFC—Agenda For Change is the current National Health Service (NHS) grading and pay system for NHS staff.

### 4.5 Recruitment

RNs meeting the inclusion and exclusion criteria were recruited through various means including NHS email, posters and internal communication. The Participant Information Sheet was embedded in emails to participants.

### 4.6 Data Collection

Hermeneutic conversation was believed only to be achievable using in‐depth interviews as a data collection tool which allowed for full exploration of the nurses' experiences and probing of their perceptions. Hermeneutic enquiry focuses on what humans experience, rather than what they know, meanings which may not be apparent but can be explored through narratives.

Interviews were undertaken from October 2019–March 2020, for an average of 32 min, starting with a single open‐ended question in line with Gadamerian underpinning principles (Gadamer 2004) to enhance reflection and commence the ‘fusion of horizons’. The goal of the interviews was to generate meaning and understanding, extracting knowledge, co‐constructing the narrative underpinned by experiences of using NEWS. Participants were encouraged to explore in‐depth their initial answer to the opening question. The researcher employed probing and discussion to thoroughly draw out participants' perceptions and experiences, with an open exchange being essential to effective data collection. The first author conducting all interviews, underpinned by her lived experience of using NEWS, was central to the success of the interviews. The length of the interviews reflected the depth of the stories shared and the positive rapport and dialogue between the two individuals. Interviews were audio recorded and transcribed verbatim by the researcher promptly after each interview, combined with field notes and reflections that were captured following each interaction.

### 4.7 Data Analysis

Data analysis followed the six‐step framework offered by Alsaigh and Coyne (2021) for hermeneutic phenomenology. These are: immersion, understanding, abstraction, synthesis and theme development, illumination and illustration of phenomena, and Integration and critique. This framework aligns to the concept that analysis and interpretation are constant from the outset of the study as demonstrated in each of the steps. Analysis was led by the first author with involvement from other authors at regular intervals to enhance study rigour and evolving understanding and new horizons.

Step 1, immersion, commenced with transcription of the data, followed by a prolonged period immersed in the data, reading each transcript multiple times, revisiting the audio tapes to explore the context, questioning the text to gain further understanding of its meaning. Step 2 (understanding) focuses on deepening understanding through interpretation, involving coding the data, questioning the text, analysing the written content, vocal nuances from audiotapes, and field notes to evolve interpretations beyond mere description.

Step 3, abstraction, involves the development of researcher constructs, through a manual process to group codes into categories and explore the verbatim quotes for other meaning. This is followed by synthesis and theme development (Step 4), integrating participants' narratives to identify common experiences and unique insights, with a meshing of horizons (Alsaigh and Coyne 2021).

The fifth step of Illumination and illustration of phenomena involved the linking of the themes to the literature and analysis of inter‐relationships, re‐visiting the literature and returning to the text for further analysis, which is not common to most research methodologies that advocate completion of the literature review prior to data collection. The purpose of reviewing the literature within the analysis stage is as a dialogical partner to provoke thinking and sits naturally in the interpretative stage of working with the data. The final step of the analysis (Step 6) refers to integration and critique focused on presentation of the research findings.

### 4.8 Ethical Considerations

The research was guided by World Health Organisation Ethical Guidelines and the International Council of Nurses (ICN) Code of Ethics for nurses. Approval was obtained from the Health Research Authority (IRAS Ref: 255031) and London South Bank University Research Ethics Committee (Ref: ETH1819‐0035).

Written informed consent was obtained prior to interview. Both confidentiality and anonymity were protected throughout the study, with collection of personal data limited to simple demographic data, such as years since registration as a RN, clinical area and pay grade. In recognition of the risk of intrusion associated with sharing lived experience, participants could stop the interviews and withdraw at any point and debriefing was made available following interview. At all stages of the research, regular conversation was held with the project sponsor and gatekeeper in the participating hospital.

### 4.9 Rigour and Reflexivity

Whilst Gadamer (2004) suggests that the hermeneutic experience, through uninterrupted listening holds its own rigour, phenomenology is frequently criticised in respect of trustworthiness. Like other qualitative methodologies, hermeneutic phenomenology requires a researcher to demonstrate a depth of rigour which captures their thoughts and understanding of meaning at each step of the research process. Tracy and Hinrichs (2017) ‘big tent’ criteria, an eight‐step conceptualisation assessing the quality of qualitative research, was applied to ensure trustworthiness in the present study. Adherence to the EQUATOR guideline, SRQR, was implemented to strengthen methodological rigour and ensure comprehensive reporting.

## 5 Findings

### 5.1 Characteristics of Participants

Sixteen RNs participated; their demographics presented in Table 2. Half of the sample were overseas nurses which is broadly representative of the NHS in London, where 66% of nurses are of British origin (Baker 2020). Despite most of these overseas nurses having many years' experiences, their exposure to NEWS was limited to the U.K., which became an important factor in their responses.

### 5.2 The Story of Using NEWS

Findings are presented in the form of a story of nurses using NEWS based on interpretation of their experiences and perceptions. Three distinct roles of the nursing team were identified (Table 3) that operate within the use of NEWS: the Health Care Assistant (HCA) a member of the unregistered workforce; the junior RN; and the senior RN.

**TABLE 3:** Nursing roles in the use of NEWS.

Role	Pay grade	Responsibility in NEWS
Health Care assistant	2–4	Undertaking vital signs Escalating Vital signs to Junior RNs
Junior RN (Jnr RN)	5	Receipt of vital signs from HCA OR undertakes vital signs Calculates NEWS Escalates concern to Senior RN based on NEWS and/or concern Calls CCOT based on response from Snr RN Stands down from patient once help achieved and responsibility shifted
Senior RN (Snr RN) – Ward Based	6‐8a	Receives escalation from Jnr RN Either takes over care of patient OR instructs Jnr RN to call CCOT Undertakes systematic assessment of patient Self–manages patient OR escalates to the medical team

**FIGURE 1:**
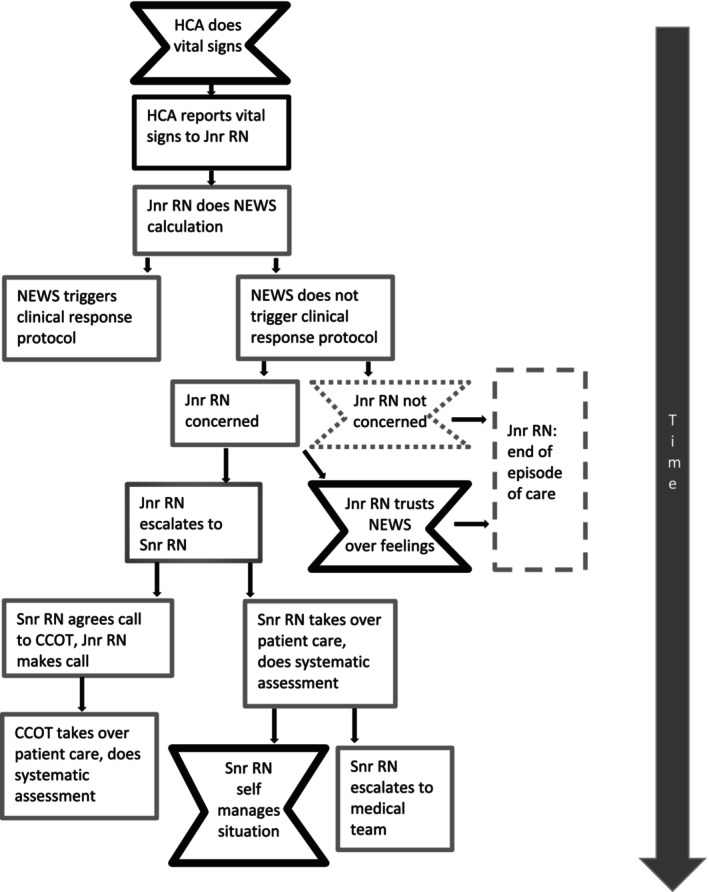
Story of NEWS with pinch points.

As the story emerged three points of risk in clinical practice were revealed (Figure 1), presented as pinch points that reflect a potential turning point in the story where an antagonistic force works against the desired goal. The pinch points represent potential patient safety failures where there is a risk that a patient's deterioration might go unnoticed or be inadequately addressed, which could ultimately result in a preventable death.

The three pinch points were identified as:

- Pinch Point 1—HCA does vital signs.
- Pinch Point 2—The junior RN trusts NEWS over feelings.
- Pinch Point 3—The senior RN self‐manages the situation alone.

#### 5.2.1 Pinch Point 1—HCA Does Vital Signs

At the start of the process of doing NEWS, and at the lowest level of the traditional nursing hierarchy, is the unregistered nursing workforce, usually referred to as HCAs. Whilst HCAs were not interviewed in the study, their involvement in the use of NEWS was frequently discussed during data collection from the perspective and experiences of the RNs. The first pinch point revealed the impact of vital signs being undertaken by HCAs. This delegation of practice created several risks which revolved around traditional practices (‘doing the ‘obs’ round’), concerns over compliance, competence, honesty and trust, impact of hierarchy, appropriate delegation, and delays to escalation.

Particular benefits resulting from the introduction of NEWS were perceived for the HCA workforce who, it was reported, undertook most of the vital sign monitoring yet received less training on vital sign interpretation. NEWS was perceived to offer a counterbalancing of the lack of training for HCAs, with a perception that less reliance needed to be placed upon the ability of the HCA to interpret patient trends in vital signs to identify changes in the patient.

I think it was useful for everyone but particularly probably useful for the HCAs because they don't have that level of training to see where a trend is happening and where something is going the right or the wrong way (N7)

Participants highlighted an underlying culture of vital sign monitoring that had not changed following NEWS implementation. ‘Doing the obs’ referred to the traditional task of undertaking clinical observations or vital signs recording at set periods and times throughout the day irrespective of the needs of the patient or the demands prescribed by the NEWS clinical response protocol. This practice was perceived to cause delays in escalation when RNs were not alerted to abnormalities until the end of the ‘obs round’ of up to 10 patients.

They do all ten patients, and that can take them an hour, and by that point, you've had that patient NEWS score in for an hour, and then they report all of the patients' Obs back to the nurse, all together. (N5)

As a result of this ‘failure to notice’ by the HCA, the RN remains unaware of potential deterioration. ‘Obs rounds’ echo task‐focused care, outdated since the 1970s in favour of more patient‐centred approaches (Langkjaer et al. 2023). Despite the NEWS protocol advocating personalised vital sign monitoring, a residual culture of recording vital signs at set times has not changed.

When a bank HCA works in the morning and then I come in at night and then a regular HCA that works on the ward comes in at night and counts the respiratory rate [and] it's really different from the previous one. So, we wonder if the previous one is really counting because the patient's already on oxygen, you know, and you can see from her that she has high respiratory rate. So, you wonder if the previous one really did it but I can't check. (N16)

Another practice issue highlighted related to compliance regarding vital sign monitoring, with recording of the Respiratory Rate (RR) being the most frequently cited throughout the dialogue. RR was raised as an ongoing frustration that existed prior to the introduction of NEWS, with participants citing incompliance, poor accuracy, and questions over the honesty of healthcare professionals taking the measurement, suggesting that people frequently falsified the RR. This was not just in relation to HCAs but the wider workforce.

People, they don't count respiratory rate… They don't, it is just … you can see the patient catching his breath when you look at obs, RR is 20… So, I think laziness, yes. Culture. (N8)

RNs were aware that people falsify the RR and, whilst it appeared to cause angst, participants indicated that there was little action taken to address it. The dialogue with RNs in this study revealed a professionally unacceptable, unquestioning acceptance of this poor practice. The reason for delegation was primarily related to RN workload.

### 5.3 Pinch Point 2—The Junior RN Trusts NEWS Over Feelings

Effective decision‐making is dependent upon clinical judgement (Tanner 2006) with the nurse interpreting signs and symptoms of deterioration and responding appropriately. The development of clinical judgement skills is relative to experience of the nurse, as is evident in the findings of this study. The second pinch point reflects concerns that junior RNs may unquestioningly rely solely on NEWS ignoring any disquiet they may feel about the patient. Consequently, if NEWS is not specifying deterioration, it may go either unnoticed or ignored.

Junior RNs, including overseas nurses, characterised rule‐following behaviour when discussing NEWS, highlighted by the language that denoted an obligation to do something or take action, based on a set of rules or permissions.

So, if I can see that the patient is deteriorating or really he's not well then I do what the NEWS scoring said to do. Then I escalate to the doctors and nurses in charge as well, so they will know and yes…and if the patient is not that, he's scoring but is not presenting symptoms, then maybe we can just observe him but not that frequent. (N1)

Explanation offered for such behaviour was concern over potential reprimand from senior colleagues,

I keep thinking that I'll get in trouble if I don't do it, because I think they [Outreach] feedback it as well, to our manager and stuff… (N14)

One senior RN suggested that following rules negated the need for making decisions and therefore as a newly qualified nurse rule‐following may be the easiest option.

… whether it's a verbal instruction or a written instruction, you're still following an instruction, therefore you're not really making decisions… –I can understand why, as a newly qualified nurse. Why would you go against the rules? (N5)

NEWS is viewed as a task which is mandated, monitored through audits and actions. Newly qualified nurses indicated that they wanted to fit in, to be part of the team which may further enforce rule‐following behaviours as if they feel that is expected of them. Concerns were highlighted with the perception that some junior RNs took an uncritical approach to NEWS, following its clinical response protocol strictly.

Junior RN participants described experiences labelled as false negatives when using NEWS. This refers to instances where NEWS does not trigger the clinical response protocol, but the nurse believes that the patient is unwell. For inexperienced junior RNs, who held less confidence and self‐belief in their skills, this led to an internal conflict as to whether they should follow the clinical response protocol or their instinct.

I've had lots of times where I've, like, looked at my patient and they're not okay but the NEWS is zero or something and the doctor's like, ‘Oh but the NEWS is zero’. (N9)

In addition to pinch point 2, there is an additional area of potential risk (identified as pinch point with a dotted line) in Figure 1, which relates to a negative trigger, where the NEWS does not trigger action and junior RN may not be concerned and therefore does not take any further action. This highlights a risk associated with trusting NEWS, that not all deteriorating patients have abnormal vital signs and less experienced nurses may not recognise the more subtle signs of deterioration or consequently may not take action.

…so you can carry on having a NEWS deteriorating can't you, so it might start at zero and then go to two and then go to three, you can have that but they don't escalate it until it gets to five which is what the trust policy says (N7)

At this point the Junior RN may not be concerned however, bearing in mind the need for clinical judgement beyond NEWS, a senior RN might have a different perspective on that patient. Whilst a triggering score encouraged junior RNs in this study to seek help, a non‐triggering score was sometimes met with uncertainty.

Senior RNs postulated that the rule‐following behaviour described was detrimental to the practice of nursing which required the process of critical thinking and clinical judgement. This perception was accompanied by concern for the future direction of the nursing profession and the skills of nurses, suggesting that unless nurses demonstrate their ability to undertake clinical judgement and work autonomously then they may be replaceable by less qualified workers.

And we're doing ourselves out of a job if we don't start to think about what is actually wrong with this person. We should have the skills… we might not necessarily know and might not necessarily get it right, but we should be thinking, ‘Okay, this person had surgery three days ago, they've been fine and suddenly they're not, what could it be?’ (N4)

Junior RNs in this study did not discuss the process of undertaking further objective systematic assessments but focused on vital signs only. This was despite training on the Airway, Breathing, Circulation, Disability, Exposure (ABCDE) assessment being highlighted by senior nursing staff on the structured assessment tool advocated universally for the assessment and treatment of critically ill patients.

[ABCDE] I teach it all the time, all the time! We teach it in everything we do… But, when they come to training and they start off doing assessments, they go straight for blood pressure. … I think they go away with the intention of doing it, but no it doesn't always transpire, so this is where I think classroom teaching is one element, but then we have got to get out there and do it, and that is what we try to do. (N6)

Within the interviews, senior RNs were cited frequently to be the first point of contact for junior RN escalation, irrespective of clinical response protocol requirements. Junior RNs seek support and reassurance from their senior colleagues who they consider more capable in dealing with complexity, before any further escalation to the Critical Care Outreach or medical team. Central to this was the accompanying shift of responsibility for the patient, allowing the junior RN to focus on their other patient caseload.

I think it's definitely easier to escalate to someone because if I see my daily work when I have ten patients and one is about to get sick because he's scoring 5 or 6 or whatever, if I escalate … How can I say this without sounding inappropriate? So, let's say if I escalate it's going to be someone else's problem because another team will come up and I will have help instead of doing everything by myself. (N12)

Once escalation took place, there was a sense of ‘passing the buck’ that occurred when a senior person arrived. This was reflected both by the junior RNs who admitted relinquishment of responsibility and the senior RNs who accepted it. This was further supported by the process of documentation, undertaken by the junior RNs, signifying the end of their input in that episode of care at the point that the senior person arrived.

I'll put add comments or flag with comments that you'll say, ‘Given water for low blood pressure, elevated legs, asymptomatic and then escalated to the doctor’. Just to back myself up. (N14)

The CCOT featured widely throughout the dialogue with the narrative portraying a high level of respect for the CCOT who were seen as a supportive ‘big brother’. The junior RN needed (and admired) the CCOT for their skills and ability to deal with the deteriorating patient but also for the power associated with their decision‐making.

I find it easier to escalate to the Outreach because I think it's easier to escalate to nurses; they're more willing to listen and they understand your worry because they're nurses as well and they've been in my shoes, they've probably been in the same position as me once so they're more understanding. But the doctors, sometimes they don't worry, they don't have the same worries as us, and something we're worried about they're probably… ‘Oh it's nothing’. (N15)

CCOT acted as the surveillance arm of the NEWS system, who surveyed the wards from a central monitoring function remotely overseeing the actions of the ward nurses.

However, even if you don't escalate, when they see the NEWS score is high the Outreach usually phone the ward, ‘Are you okay? Is this patient okay?’ (N10)

RNs described incidents where CCOT contacted them to check up on them when a patient triggered the electronic NEWS system. This gave the junior RNs a sense of security, viewed as supportive and helpful rather than invasive. Dealing with an acutely unwell patient was recognised as challenging and complex so knowing that someone else was monitoring patients remotely gave nurses reassurance.

### 5.4 Pinch Point 3—The Senior RN Self‐Manages the Situation

Whilst junior RNs were perceived to have greater dependence on NEWS and subsequent escalation, it became clear that senior RNs were less likely to escalate, in particular to CCOT, when the NEWS escalation protocol indicated this. Senior RNs shared instances where they requested the Critical Care Outreach Team (CCOT) to “stand down” in response to an escalated NEWS score, before the team even arrived on the ward. Senior RN participants likened members of the CCOT to their own level of competence and ability.

We know why the patient's like that, we know why they're triggering that, and we are managing it, they're on the treatment, they're on the right antibiotics, they're on oxygen, the humidifiers, they're on those, that is the treatment. What else could Outreach do that we can't do? (N4)

Whilst senior RNs in this study were less reliant on NEWS, they also applied the NEWS protocol flexibly where scores were low. This decision stemmed from the application of their clinical judgement, where they chose not to escalate but instead to initiate and monitor specific interventions. They described instances where, based on their assessment, immediate escalation was unnecessary, and carefully chosen interventions could be evaluated for effectiveness before taking further steps.

Yeah, but then it just has to go alongside clinical judgement like you can't just use it as a tool all on its own because that doesn't work. (N9)

There were experiences where senior RNs operated outside of NEWS by advocating in the best interests of the patient as opposed to following a protocol.

I just stood there and I went, ‘I'm really sorry but this lady's dying, can we just get her husband in and let him have a last few minutes with her?’ And the junior doctors apparently felt undermined by me. We couldn't get a blood pressure on her, we could barely get a pulse so I just felt that again nobody was looking at the patient to look at what was happening (N7)

This further demonstrates the application of critical thinking skills in senior RNs who valued a patient and family centred approach above compliance with NEWS when they believed this to be appropriate.

## 6 Discussion

The use of NEWS is well established and is recognised in the U.K. and internationally as having an important role in the recognition and management of patient deterioration. However, this study has identified that when NEWS is used, there is potential for any of three pinch points to affect the course of action. Making the wrong judgement at a single pinch point could be detrimental to the safety of patients. It is therefore reasonable to assume that errors in clinical judgement at all three pinch points in the care of an individual patient could negatively impact patient outcomes, with the potential for death or other serious adverse events.

The first pinch point revealed the risks associated with the delegation tasks associated with using NEWS to the healthcare assistant workforce, linked to the RN workload. Other studies have identified similar risks, noting the potential loss of critical information necessary for clinical judgement when NEWS responsibilities are delegated to less experienced staff without assurance of competence beyond recording vital signs (Langkjaer et al. 2023). Delegation of vital signs monitoring to HCAs also was linked to delays in escalation, reflecting immersion in a traditional culture of ‘obs rounds’, task‐orientated approaches to care. This non‐patient centred behaviour may be driven by perceived efficiency gains (Dall'Ora et al. 2021) which are not compliant with EWS clinical response protocols and may represent a missed opportunity to detect clinical deterioration with potential serious outcomes for the patient.

Competing beliefs about the need for RNs to delegate monitoring to HCAs are cited within the literature with some RNs suggesting delegation and oversight of HCAs was part of the RN role whilst others believed that the HCA should inherently know when to enact the required behaviours (Smith et al. 2021). Delegating, despite knowing that an HCA is unlikely to complete the task as required, places the RN potentially in breach of their professional standard.

Internationally, the education and training of support workers is poorly governed, with the development of regulation of this crucial, yet invisible, workforce seen as imperative across the globe (Saks 2021). There is no benchmark or national competence for HCAs with regards to vital sign monitoring or the interpretation required to act upon deterioration. For example, in U.K., the Care Certificate is a nationally recognised competence‐based certificate yet fails to include skills around deterioration recognition.

Junior nursing staff demonstrate a reliance on NEWS which offers them a reassurance, a safety net (Ferguson, Baldwin, and Henderson 2024; Massey et al. 2024), a sense of professional control and protection, reflecting a comprehensive standard of care that is less subject to risk and criticism. In turn, this allows them to ‘cover their back’ on the basis that professional judgement may involve risks of wrong assessment and criticism. Reliance on a checklist like NEWS encourages rule‐following behaviour, as this study demonstrates. Whilst rule‐following is vital in some aspects of nursing, for the development of the advanced beginner (Benner 2001); in the absence of experts there is a risk that rules will take precedence over expertise. Phenomenologist Heidegger warned of a way of thinking and acting that is overtaken by protocol‐driven environments where people fail to think (Heidegger 1962). He suggested that while both measurement and calculation are essential, they can also remove an awareness of experience and have a de‐skilling effect. NEWS was not intended to be used in this skills‐inhibiting way but as a tool to aid decision‐making, not replace clinical judgement (RCP, 2017).

Application of the four stages of clinical judgement (Tanner 2006) to the findings of this study revealed that the junior RN ‘notices’ the patient, escalates to raise the alarm but does not use their tools and knowledge to provide an immediate response, instead they wait for a senior RN to take action. Tanner (2006) identifies that less‐experienced nurses often lack the knowledge and experience to fully undertake even the stage of noticing as they lack a frame of reference. EWS can be seen to help or hinder critical thinking with some studies demonstrating a perceived value of EWS in initiating critical thinking (Le Lagadec et al. 2024) and supporting risk assessment (Langkjaer et al. 2023) whilst others highlight the frustration associated with EWS when the score does not align to nurses critical thinking (Massey et al. 2024).

These findings highlight a risk that the behaviour associated with the use of NEWS may lead to the nursing profession losing important problem‐solving and critical thinking skills. Continuation of this behaviour presents a picture emulated by dependency on objective data without consideration of the clinical picture, with a subsequent impact on the development of critical thinking skills in novice nurses (Massey et al. 2024). With junior nurses keen to escalate to supportive senior nursing staff (Langkjaer et al. 2021) there is a shift of responsibility to the senior RN. This reflects one of the five principles of a High Reliability Organisation (HRO), labelled as a ‘deference to expertise’ where during a crisis, decisions are made by the person with the skills, knowledge and experience to solve the problem. HROs are organisations that operate in complex, high‐risk environments yet experience fewer than anticipated accidents or harm events (Health and Safety Executive 2011). However, one disadvantage of this approach is the lack of skills acquisition in decision‐making for the junior workforce, further compounding the development of clinical judgement.

For more experienced nurses, there is a tension between EWS and critical thinking skills, which can lead to ‘white lies’ (Massey et al. 2024) with nurses manipulating EWS to align with their own clinical judgement, rejecting the rigid nature of the tool and its depersonalisation of patient care (Ferguson, Baldwin, and Henderson 2024). Other studies (Langkjaer et al. 2021) discuss the inflexibility of NEWS leading to nurses modifying scores, deviating from escalation protocols and the frustration associated with this, with nurses admitting to the omission of some vital signs the perceived unnecessary (Le Lagadec et al. 2024). More experienced nurses use NEWS as an adjunct to their decision‐making, applying the tool flexibly and not escalating as per the guidance underpinned by their confidence in self‐managing the situation.

Since its release, the RCP has fiercely defended criticisms of NEWS referring to its successes from evaluative data. However, the quality of these data is reported to be poor with identification of poor methods and inadequate reporting across 95 published studies (Gerry et al. 2020). One such criticism is that NEWS has become a replacement for, rather than an adjunct to, clinical judgement as intended (Ferguson, Baldwin, and Henderson 2024). Prior to NEWS, clinical judgement alone proved insufficient for detection of deterioration with evidence of continuing failure to identify and properly manage patient deterioration (RCP, 2017). However, the implementation of NEWS has not eradicated the phenomenon of missed patient deterioration.

### 6.1 Strengths and Limitations

The major strength of this study is its focus on nurses as the main users of NEWS, exploring their experiences and perceptions on an individual basis to gain deeper understanding of the phenomena. The study sample was diverse in experience, clinical speciality and the setting was typical of an acute hospital with detail provided to aid the perceived transferability of the findings.

The study focuses on a highly topical patient safety issue that has national and international relevance issue and poses a challenge in healthcare delivery. This study makes a significant contribution to the evolving evidence base around the deteriorating patient phenomenon specifically focusing on nurses use of NEWS with a focus on clinical judgement.

This study commenced prior to the COVID‐19 pandemic and the authors acknowledge this may not necessarily reflect the nurses' experience of NEWS post pandemic. Furthermore, the study was limited to data collected from RN staff. Findings may therefore not reflect the perceptions of other members of the nursing team, such as the unregistered workforce, who feature in the use of NEWS but excluded from inclusion in the study.

Despite some identified limitations, the use of hermeneutic phenomenology, which is uncommon in nursing research, in particular for patient safety research, provided unique insights into the experiences of nurses. The fusion of horizons enabled shared meaning of experiences resulting in an enhanced understanding of risks associated with the use of EWS.

### 6.2 Recommendations for Further Research

Future research should focus on exploring the use of EWS (both NEWS and other tools) within the wider nursing workforce, including the unregistered nurse workforce, as well as the experiences of the CCOT. Further exploration of decision‐making processes when EWS does not trigger would contribute to greater understanding of the extent of clinical judgement and reasoning process that junior RNs make when EWS does not reflect a nurses' developing clinical intuition. Furthermore, understanding the actions of senior RNs as the first point of contact when a patient deteriorates through observational studies would support data from this study which focuses on perceptions.

## 7 Conclusion

EWS were introduced into clinical practice as an aid to clinical assessment, to supplement clinical judgement in acute care, not to substitute for competent clinical judgement. Taking a hermeneutic phenomenological approach, this study allowed nurses' experiences and perceptions to be thoroughly explored with the lead researcher transposing herself into the participants' horizon to elicit meaning from these experiences.

The study is embedded in patient safety practice, so the response to its findings should focus on minimising the risks identified at each of the three pinch points. HCAs are an invaluable group of staff who require urgent investment to develop knowledge, skills and attributes required for identification of early signs of deterioration such as behavioural changes, alongside monitoring and interpreting vital signs, and assimilation of escalation skills. When delegating vital signs monitoring, the RN must be assured that the recipient holds the necessary competence, irrespective of their role or position within the workforce. RNs should be reminded of their professional and legal responsibility for delegation through their NMC registration.

Junior RNs should endeavour to engage with the four stages of Tanner's clinical judgement model when using EWS. Encouraging a workplace culture which supports all nurses to engage with the four stages of Tanner's clinical judgement model whenever they are carrying out NEWS offers potential to save lives, developing and empowering nurses to make appropriate clinical decisions. Education to underpin the development of clinical judgement skills should be available, potentially utilising simulation‐based education and underpinned by evidence‐informed competency frameworks.

## Author Contributions

C.N., S.B., Y.H., L.T.: made substantial contributions to conception and design, or acquisition of data, or analysis and interpretation of data. Involved in drafting the manuscript or revising it critically for important intellectual content. Given final approval of the version to be published. Each author should have participated sufficiently in the work to take public responsibility for appropriate portions of the content. C.N.: agreed to be accountable for all aspects of the work in ensuring that questions related to the accuracy or integrity of any part of the work are appropriately investigated and resolved.

## Conflicts of Interest

The authors declare no conflicts of interest.

## Peer Review

The peer review history for this article is available at https://www.webofscience.com/api/gateway/wos/peer‐review/10.1111/jan.16656.

## Data Availability

The data that support the findings of this study are available on request from the corresponding author. The data are not publicly available due to privacy or ethical restrictions.
